# Supplementary material for: Hepatitis B Virus DNA is a Substrate for the cGAS/STING Pathway but is not Sensed in Infected Hepatocytes
Source: Viruses. 2020 May 29;12(6):592. doi: 10.3390/v12060592 (PMC7354540; doi:10.3390/v12060592)
Supplement: Supplementary file 1 [file viruses-12-00592-s001.pdf]

viruses

Supplementary Figures for

Hepatitis B virus DNA is a Substrate for the cGAS/STING Pathway but is not Sensed in Infected Hepatocytes

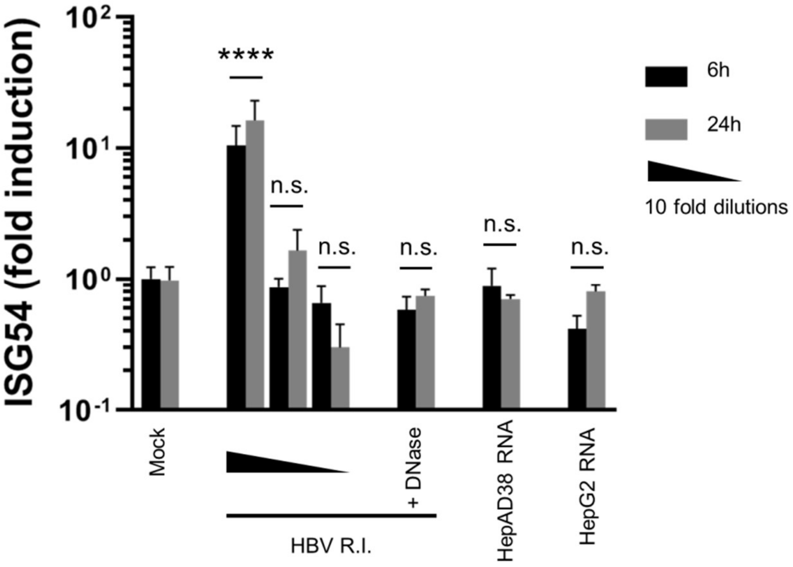

Figure S1: Two-time-point analysis of the immunostimulatory potential of viral nucleic acids in MDDCs. For the indicated viral nucleic acids, the induction of ISG54 mRNA was analyzed at 6h and 24h post transfection in two of the MDDC donors shown in Figure 1. Levels of significance compared to mock were determined using a Mixed effect analysis of 6h + 24h with Dunnett's multiple comparisons test (****$p < 0.0001$, n.s.: not statistically significant). Average and SEM of 3 technical replicates of 2 donors are shown. HBV R.I.: HBV replication intermediates.

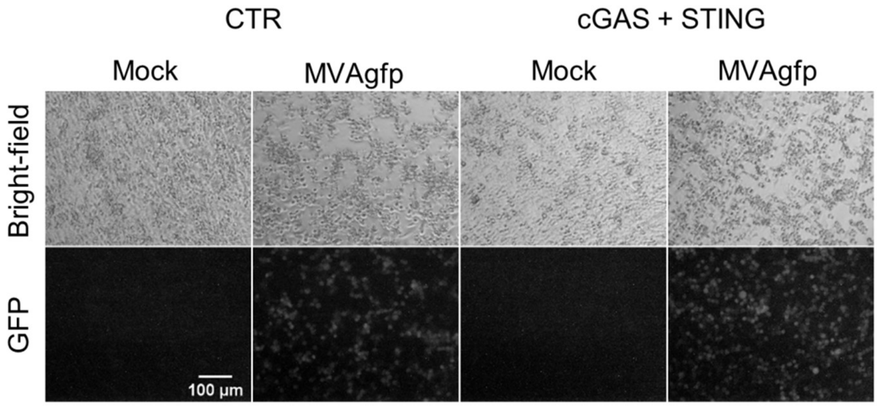

Figure S2: Infection control for MVA-gfp infection of HepG2-hNTCP. Gfp expression in the MVA-gfp-infected HepG2-hNTCP CTR or cGAS+STING cells used in Figure.4, 20 h post infection.

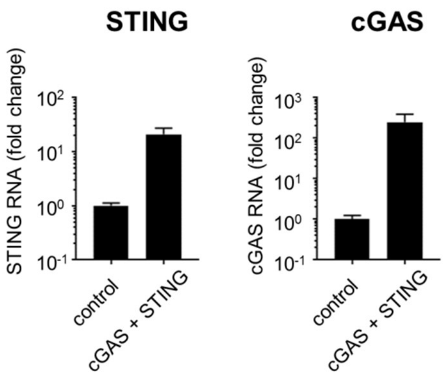

Figure S3: cGAS and STING overexpression in HepG2-hNTCP. The expression level of cGAS and STING mRNA in HepG2-hNTCP transduced with a control lentiviral vector or with lentiviral vectors expressing cGAS and STING used in Figure 4B was determined by RT-qPCR. The graph shows the average and SEM of 2 experiments performed in technical triplicates.

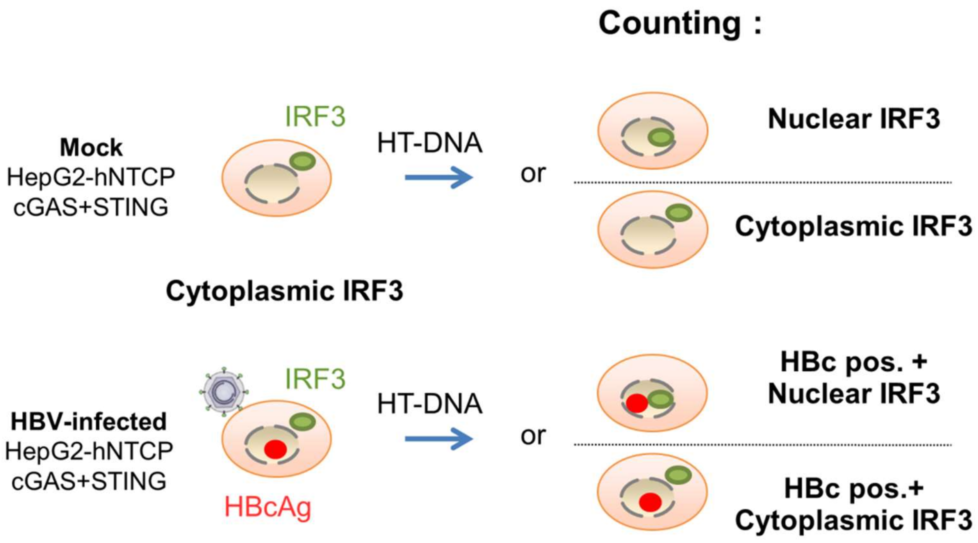

Figure S4: Experimental design for Figure 6. HepG2-hNTCP overexpressing cGAS and STING were infected or not with HBVwt (MOI 10000). 7 dpi, the cells were transfected with HT-DNA to stimulate the DNA sensing pathway. 16 hours post transfection the cells were fixed and immunostained for IRF3 (Alexa 488, green), HBc (Alexa 555, red) and DNA (Hoechst, blue). The percentage of cells harboring a nuclear staining for IRF3 in the mock-infected cells after HT-DNA transfection or in the HBc positive cells from the HBV-infected and HT-DNA-transfected samples was counted and is indicated in the graph Figure 6B.

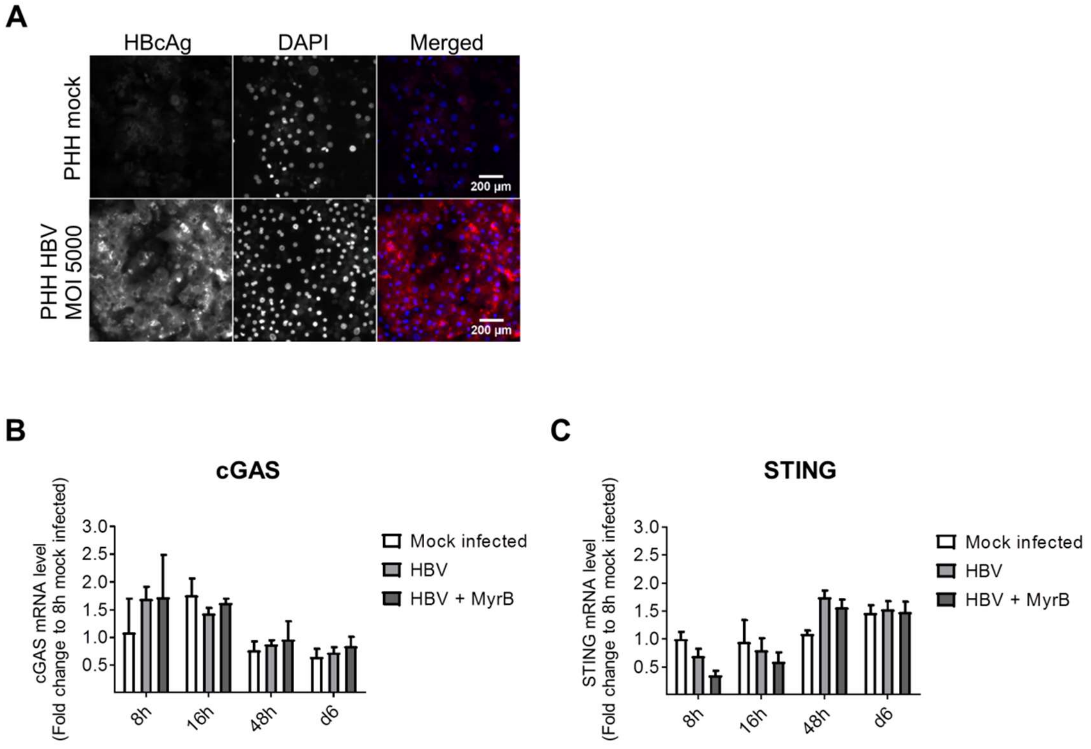

Figure S5: HBV does not affect the expression level of cGAS or STING. PHH were infected with HBVwt (MOI 5000). (A) Immunofluorescence staining of HBcAg (red) in mock- or HBV-infected PHH 7 days post infection. cGAS (B) or STING (C) mRNA levels were quantified by RT-qPCR at the indicated times post infection. The HBV entry inhibitor Myrcludex B (MyrB; 1µM) was added as a control. The graph represents the average and standard error of the mean (SEM) of 3 technical replicates of at least 2 donors.
